# The ClC-2 Chloride Channel Activator, Lubiprostone, Improves Intestinal Barrier Function in Biopsies from Crohn’s Disease but Not Ulcerative Colitis Patients

**DOI:** 10.3390/pharmaceutics15030811

**Published:** 2023-03-02

**Authors:** Young Su Park, Sang Bum Kang, Ronald R. Marchelletta, Harrison M. Penrose, Roos Ruiter-Visser, Barbara Jung, Michael J. Docherty, Brigid S. Boland, William J. Sandborn, Declan F. McCole

**Affiliations:** 1 Division of Gastroenterology, School of Medicine, University of California San Diego, La Jolla, CA 92093, USA; 2 Department of Internal Medicine, Division of Gastroenterology, Seoul National University College of Medicine, Seoul 03080, Republic of Korea; 3 Division of Gastroenterology, Department of Internal Medicine, St. Mary’s Hospital, Catholic University of Korea, Seoul 06591, Republic of Korea; 4 Department of Internal Medicine, Radboud University Medical Center, 6525 GA Nijmegen, The Netherlands; 5 Division of Biomedical Sciences, School of Medicine, University of California, Riverside, 307 SOM Research Building, 900 University Ave, Riverside, CA 92521, USA

**Keywords:** chloride secretion, epithelial, inflammatory bowel disease, ion transport, occludin, permeability, tight junction

## Abstract

The prostone analog, lubiprostone, is approved to manage constipation-predominant irritable bowel syndrome. Lubiprostone also protects intestinal mucosal barrier function in animal models of colitis. The aim of this study was to determine if lubiprostone improves barrier properties in isolated colonic biopsies from Crohn’s disease (CD) and ulcerative colitis (UC) patients. Sigmoid colon biopsies from healthy subjects, CD and UC patients in remission, and CD patients with active disease were mounted in Ussing chambers. Tissues were treated with lubiprostone or vehicle to determine the effects on transepithelial electrical resistance (TER), FITC-dextran 4kD (FD4) permeability, and electrogenic ion transport responses to forskolin and carbachol. Localization of the tight junction protein, occludin, was determined by immunofluorescence. Lubiprostone significantly increased ion transport across control, CD and UC remission biopsies but not active CD. Lubiprostone selectively improved TER in both CD remission and active disease biopsies but not in control or UC biopsies. The improved TER was associated with increased membrane localization of occludin. Lubiprostone selectively improved barrier properties of biopsies from CD patients vs. UC and independent of an ion transport response. These data indicate that lubiprostone has potential efficacy in improving mucosal integrity in Crohn’s disease.

## 1. Introduction

The current mainstay of IBD treatment is the use of broad-spectrum anti-inflammatory drugs, but there are several complications and limitations with this approach including the lack of effect in rectifying an underlying permeability defect in the intestinal epithelium that permits excessive access of luminal antigens to the lamina propria. Lubiprostone (Amitiza^TM^; Takeda Pharmaceuticals Inc.), a bicyclic fatty acid prostone derivative, was developed to enhance intestinal fluid secretion in treating constipation. Lubiprostone activates luminally directed chloride secretion in intestinal epithelial cells (IEC), thereby increasing luminal fluid content and stool hydration. This alleviates symptoms associated with irritable bowel syndrome associated with constipation (IBS-C) and chronic idiopathic constipation (CIC) [1,2,3,4]. Initial studies indicated that lubiprostone could directly bind to, and activate, ClC-2 in intestinal epithelial cells (IEC) and this was supported by recent in vitro evidence of a fatty acid binding site on ClC-2 expressed in human IEC lines that functionally mediated lubiprostone-stimulated ion transport independent of the cystic fibrosis transmembrane conductance regulator (CFTR) or the prostaglandin E receptor 4 (EP4) [5,6,7,8,9]. However, this proposed mechanism of specific ClC-2-mediated Cl^-^ secretion has proven controversial as a number of conflicting studies have indicated a role for the CFTR chloride channel in the overall ion transport and Cl- secretory response to lubiprostone using human IEC lines, isolated human intestinal tissue and enteroids generated from CFTR knockout mice and cystic fibrosis patients harboring the DF508 mutation that prevents appropriate CFTR trafficking to the epithelial apical membrane [5,6,8,10,11,12,13]. An additional study in an inducible mouse model of ClC2 in nasal epithelium identified that lubiprostone could activate both ClC2 and CFTR [14].

While much of the attention on lubiprostone has focused on its ability to promote fluid secretion to alleviate constipation–regardless of the precise mechanism of chloride secretion—this agent has also demonstrated efficacy in modulating other aspects of intestinal epithelial function [15,16]. Lubiprostone has been shown to exert a protective effect on the intestinal mucosal barrier function through ClC-2 activation. Studies by Moeser et al. demonstrated that lubiprostone stimulated rapid repair of intestinal barrier function in ischemic-injured porcine ileum, while lubiprostone has also shown efficacy in alleviating symptoms of experimental colitis and NSAID-induced injury in rodents and humans [17,18,19,20,21]. The mechanism of lubiprostone efficacy was associated with activation of ClC-2 as lubiprostone treatment resulted in co-localization of ClC-2 with tight junction proteins such as occludin in the region of the apical tight junction [22,23,24]. Moreover, ClC-2 has been shown to modulate tight junction barrier function as a result of intracellular trafficking of occludin [25,26]. Confirmation of a role for ClC-2 in the restoration of functional measures of tight junction integrity were provided by studies showing that the protective effect of lubiprostone against dextran sulfate sodium (DSS) colitis, and ischemic colitis, was limited in *Clc2*^−/−^ mice [20,27,28,29].

In this study, we set out to identify if lubiprostone can improve barrier function defects associated with IBD by using explanted colonic tissue isolated from control subjects as well as Crohn’s disease (CD) and ulcerative colitis (UC) patients in remission or CD patients with active disease. We functionally characterized a beneficial effect of lubiprostone in partially improving ex vivo intestinal permeability in Crohn’s disease through modifying membrane localization of the tight junction protein occludin. These studies extrapolate the findings of a number of experimental models to human IBD and indicate a possible therapeutic potential for lubiprostone in restoring intestinal barrier function in the setting of inflammation.

## 2. Results

### 2.1. Colonic Biopsies from CD and UC Patients in Remission Have an Intact Epithelium and Display Normal Baseline Electrophysiological Parameters

**Figure 1:**
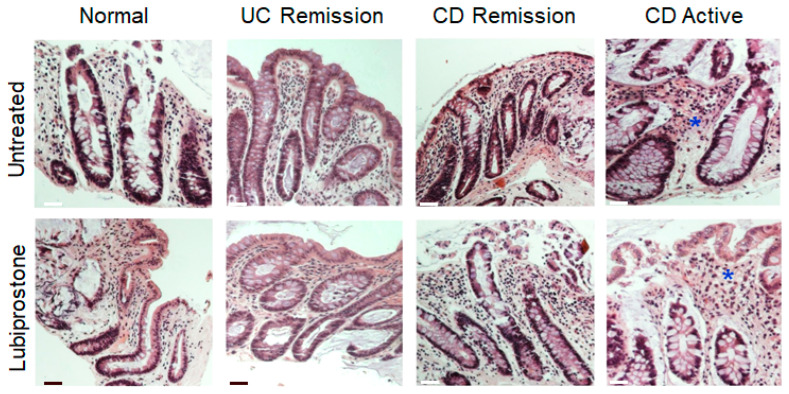
An intact epithelium is preserved in biopsies from IBD patients in remission or CD patients with active disease. Hematoxylin and eosin (H&E) staining of formalin-fixed sigmoid colon biopsies derived from control patients, patients with CD or UC in remission, and patients with active CD. Representative biopsies isolated adjacent to the healthy controls and biopsies from Crohn’s disease and UC patients in remission that were subsequently used in “untreated” vs. “lubiprostone”-treatment groups indicated no signs of significant inflammatory disease. CD patients with active disease show increased mucosal immune cell infiltrates (blue asterisk). (scale bar: 200 μm).

Given that the focus of our study was on analyzing epithelial transport and barrier properties, we first determined that colonic biopsies from CD and UC patients had an intact epithelial layer. Representative histological H&E-staining of intestinal biopsies derived from control subjects, patients with UC (in remission) and patients with CD (active vs. in remission) showed that colonic biopsies from healthy control patients and patients with Crohn’s disease or UC in remission had no signs of significant inflammation while an intact epithelium was present in all cases (Figure 1). Biopsies from patients with active CD showed more immune cell infiltrates (asterisk) as compared to control subjects and IBD patients in remission, but the epithelium was intact (Figure 1).

**Figure 2:**
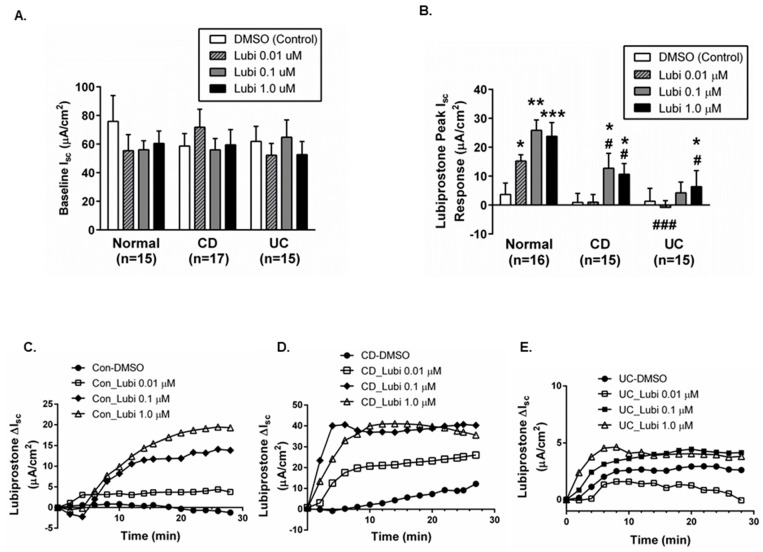
Lubiprostone stimulates ion transport across colonic biopsies from control subjects and IBD patients. (**A**) Four colonic tissues from individual normal, CD and UC subjects were mounted in Ussing chambers and basal I_sc_ measurements were recorded. No differences in baseline I_sc_ were observed between control (*n* = 15 subjects), CD (*n* = 17 subjects), or UC (*n* = 15 subjects)). (**B**) Lubiprostone increased I_sc_ in a concentration-dependent manner in all tissues (*p* < 0.05–*p* < 0.001; *n* = 15–16 subjects) compared with DMSO control treated biopsies (*n* = 14–15 subjects). However, the magnitude of response was lower in tissues from CD and UC patients vs. normal subjects (*p* < 0.05; *n* = 15). (**C**) Representative time course of the I_sc_ response to lubiprostone in control colonic biopsies. (**D**) Representative time course of the I_sc_ response to lubiprostone in Crohn’s disease colonic biopsies. (**E**) Representative time course of the I_sc_ response to lubiprostone in UC colonic biopsies. *, *p* < 0.05; **, *p* < 0.01; ***, *p* < 0.001 vs. DMSO (control). #, *p* < 0.05; ###, *p* < 0.001 vs. I_sc_ response to equivalent dose of lubiprostone in tissues from non–IBD (‘normal’) subjects.

Functional confirmation of an intact mucosal epithelium in isolated biopsies was provided by electrophysiological studies in Ussing chambers where no significant difference was observed in resting basolateral ion transport activity (measured as short-circuit current (I_sc_)) in colonic biopsies from control subjects vs. CD or UC in remission (Figure 2A).

### 2.2. Colonic Biopsies from CD and UC Patients in Remission Show Differentially Reduced ion Transport Responsiveness to Lubiprostone

We next determined if biopsies from CD and UC patients in remission were capable of exhibiting key electrophysiological responses to lubiprostone, namely an increase in electrogenic ion transport coupled with subsequent inhibition of cAMP-driven ion transport responsiveness to the adenylyl cyclase activator, forskolin. Increasing concentrations of lubiprostone induced a dose-dependent increase in ion transport responsiveness (DI_sc_), compared with vehicle control (DMSO only), across colonic biopsies from control subjects with responses reaching a peak plateau at 0.1 and 1.0 μM (Figure 2B). Biopsies from CD patients in remission also showed a significant increase in I_sc_ responses to lubiprostone but not at the lower dose of 0.01 μM. Of note, the peak responses at 0.1 and 1.0 μM were significantly lower than responses in control subject tissues (Figure 2B). In UC patient biopsies, lubiprostone only induced a significant increase in I_sc_ vs. vehicle control at the highest concentration (1.0 μM; *p* < 0.05) but this was significantly lower than the response to the same concentration in control subjects (*p* < 0.05; Figure 2B). The onset and pattern of the ion transport response to lubiprostone were similar in control, CD and UC in remission biopsies when sample traces were plotted over time (Figure 2C–E). Overall, these data indicate that ion transport responses to lubiprostone are partially suppressed in colonic tissues from CD patients in remission and further suppressed in tissues from UC patients in remission.

**Figure 3:**
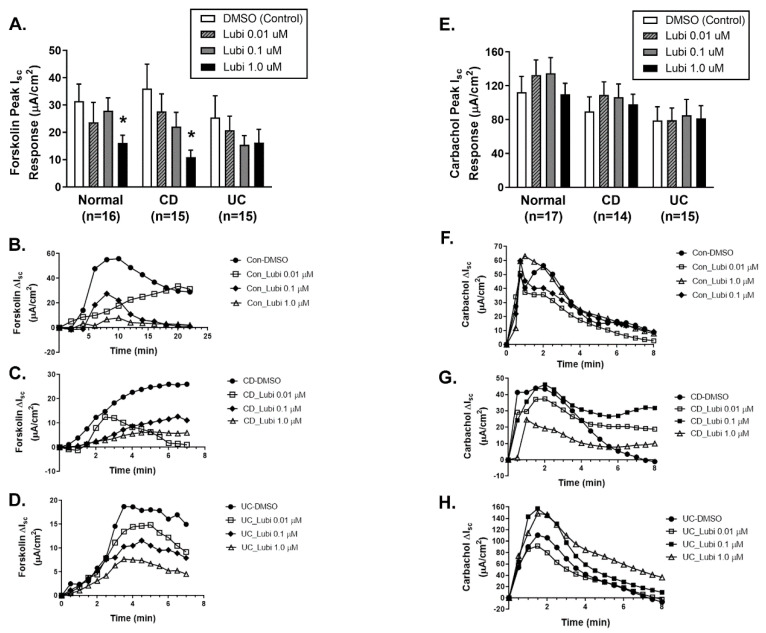
Lubiprostone inhibition of cAMP but not Ca^2+^-dependent colonic ion transport is differentially preserved in CD vs. UC. Four colonic tissues from individual normal (*n* = 16), CD (*n* = 15) and UC (*n* = 15) subjects in remission were mounted in Ussing chambers. Individual biopsies from each subject in each disease group were pre–treated with (i) DMSO–Control; (ii) 0.01 mM; (iii) 0.1 mM; or 1.0 mM Lubiprostone for 30 min before subsequent challenge with the cAMP agonist forskolin (20 μM; bilaterally, (**A**–**D**)) or the Ca^2+^-dependent agonist carbachol (CCh; 300 μM; serosally; (**E**–**H**)) and I_sc_ responses were measured. (**A**). Overall I_sc_ responses to forskolin were similar in control, CD and UC tissues, while lubiprostone reduced the I_sc_ response to forskolin in a dose-dependent manner. This effect was significant in control and CD biopsies (*p* < 0.05; *n* = 15–16). (**B**). Representative time course of the I_sc_ response to forskolin in control colonic biopsies. (**C**). Representative time course of the I_sc_ response to forskolin in Crohn’s disease colonic biopsies. (**D**). Representative time course of the I_sc_ response to forskolin in Ulcerative colitis colonic biopsies. (**E**). The I_sc_ response to CCh was similar in all three subject groups and was unaffected by lubiprostone pre-treatment (*n* = 15–17 subjects). (**F**). A representative time course of the I_sc_ response to carbachol in control colonic biopsies. (**G**). Representative time course of the I_sc_ response to carbachol in Crohn’s disease colonic biopsies. (**H**). Representative time course of the I_sc_ response to carbachol in ulcerative colitis colonic biopsies. *, *p* < 0.05 vs. DMSO (control).

A feature of lubiprostone-induced ion transport responses is subsequent inhibition of cAMP-driven Cl^-^ secretory responses to forskolin. In biopsies from control (healthy) subjects and CD patients in remission previously exposed to lubiprostone (30 min) in Ussing chambers, the magnitude of forskolin (20 μM bilaterally)-induced ion transport responses was decreased in a largely lubiprostone concentration-dependent manner. Specifically, the highest concentration of lubiprostone pre-treatment (1.0 μM) significantly inhibited subsequent responses to forskolin (*p* < 0.05; Figure 3A). Of note, the inhibitory effect of lubiprostone on forskolin-stimulated I_sc_ was not observed in tissues from UC patients in remission to be statistically significant (Figure 3A). Representative electrophysiological traces for control (Figure 3B), CD (Figure 3C) and UC (Figure 3D) are shown. To ensure that all tissues remained functionally viable and responsive to a stimulus of ion transport that is not affected by lubiprostone, tissues were challenged with the calcium-dependent acetylcholine analog, carbachol (CCh). Consistent with our previous work and studies in other model systems, lubiprostone pre-treatment had no inhibitory effect on carbachol-stimulated Ca^2+^-dependent ion transport responses in biopsies from control subjects (Figure 3E). Similarly, carbachol-stimulated ion transport responses in biopsies from CD and UC patients were unaffected by lubiprostone pre-treatment (Figure 3E). Representative ion transport responses to carbachol over time are shown for control (Figure 3F), CD (Figure 3G) and UC (Figure 3H).

### 2.3. Mucosal Barrier Responsiveness to Lubiprostone in Quiescent Crohn’s Disease

**Figure 4:**
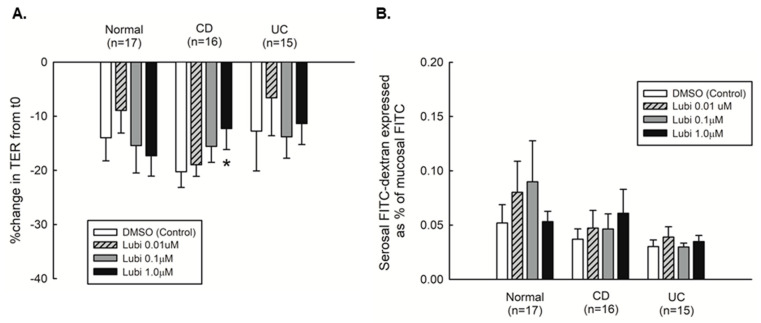
Lubiprostone selectively improves colonic mucosal barrier function in quiescent Crohn’s disease. Four colonic tissues from individual normal (*n* = 17), CD (*n* = 16) and UC (*n* = 15) subjects in remission were mounted in Ussing chambers. Individual biopsies from each subject in each disease group were treated with (i) DMSO–Control; (ii) 0.01 mM; (iii) 0.1 mM; or 1.0 mM Lubiprostone and changes in transepithelial electrical resistance (TER) and FITC-dextran 4kD (FD4) permeability were measured over two hours. (**A**). Lubiprostone increased the diminished TER in CD biopsies in a concentration-dependent manner with a peak improvement of 8 ± 3% (1.0 µM; *n* = 17, *p* < 0.05) over 120 min vs. untreated. Lubiprostone had no significant effect on UC or normal control biopsies. (**B**). FITC–dextran 4kD permeability was not significantly altered in CD or UC vs. normal controls and was unaffected by lubiprostone. *, *p* < 0.05 vs. DMSO (control).

To identify if lubiprostone affected intestinal barrier function in colonic biopsies, we mounted biopsies in Ussing chambers and probed for changes in transepithelial electrical resistance (TER) and permeability to FITC-dextran (4kD) over time (120 min). Tissues were kept in open-circuit conditions for the duration of the experiment apart from recording of individual TER measurements which were taken under closed circuit conditions. The change in TER over the course of the experiment was equivalent in tissues from control subjects, CD and UC patients in remission (Figure 4A). However, only the biopsies from CD patients responded to lubiprostone with an improvement in TER that occurred in a concentration-dependent manner, with the highest concentration (1.0 μM) causing a small but dose-dependent increase in TER vs. vehicle control (*p* < 0.05, Figure 4A). There was no significant difference in permeability to FD4 across biopsies from all patient groups and lubiprostone had no effect on FD4 permeability (Figure 4B). These data suggest that lubiprostone selectively improves TER in biopsies from CD patients in remission.

### 2.4. Lubiprostone Stimulates Occludin Relocalization in Colonic Biopsies from Crohn’s Disease Patients in Remission

Modulation of epithelial permeability is regulated by transmembrane proteins in the apical junctional complex, specifically, proteins that make up the apical tight junction. Since lubiprostone has been shown to promote occludin localization at the tight junction in ischemic porcine intestine, we focused on this tight junction protein as a molecular target that may contribute to the improved barrier performance induced by lubiprostone.

**Figure 5:**
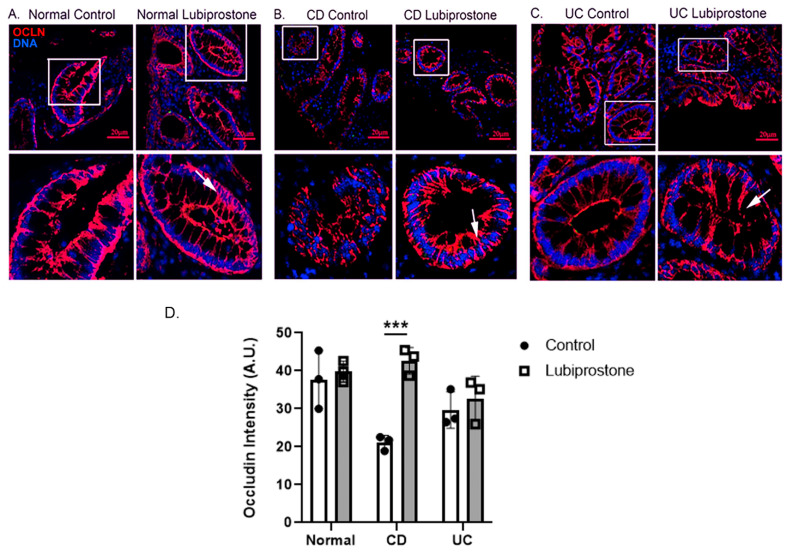
Lubiprostone increases occludin membrane localization in quiescent Crohn’s disease colonic biopsies. (**A**). Lubiprostone (1 uM; 120 min) did not appear to increase relocalization of the TJ protein occludin (OCLN; red) in colonic biopsies from normal control subjects. (**B**). However, apical and lateral localization of occludin (arrows) was increased in lubiprostone-treated CD patient biopsies vs. vehicle control CD biopsies. (**C**). In contrast, lubiprostone did not appear to increase relocalization of occludin in UC biopsies. (*n* = 4; 8 fields of view). Nuclear DNA (blue) was stained with Hoechst 33258. (**D**). Quantification of apical membrane occludin staining in intestinal epithelium. Data expressed as fluorescence intensity in arbitrary units (A.U.). ***, *p* < 0.001 vs. control.

Lubiprostone (1.0 μM; 120 min) did not appear to modulate localization of the TJ protein occludin (red) in colonic biopsies from normal control subjects (Figure 5A). However, apical and lateral localization of occludin (arrows) was increased in lubiprostone-treated CD patient biopsies vs. vehicle control CD biopsies (Figure 5B,D). In contrast, lubiprostone did not appear to increase the intensity of occludin staining in the lateral junctions of UC biopsies to the same extent as CD biopsies (Figure 5C).

### 2.5. Lubiprostone Stimulates Improved Colonic Barrier Function in Active Crohn’s Disease

**Figure 6:**
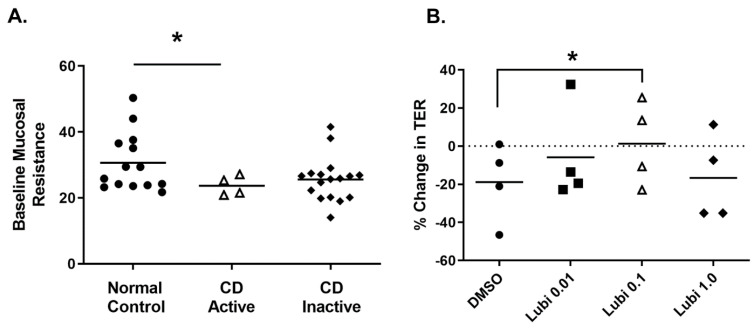
Lubiprostone improves TER in active Crohn’s disease colonic biopsies. (**A**). The baseline TER expressed in Ohms.cm^2^ (prior to addition of pharmacologic agents) in colonic biopsies from normal (non–IBD) control subjects (25 tissues from 16 different subjects) and CD patients with active disease (13 tissues from 4 different subjects) mounted in Ussing chambers was measured. Individual datapoints represent the TER value per human subject. Where multiple biopsies were used from the same subject, the mean TER value for that subject is plotted. (**B**). The change in TER over the full duration of the experiment (120 min) was assessed. Treatment with lubiprostone (0.1 μM) significantly preserved mucosal TER over time compared with vehicle control (DMSO). *, *p* < 0.05 vs. DMSO (control).

We next determined if lubiprostone could also improve TER in biopsies from patients with active CD. Biopsies from Crohn’s patients with active disease had a significantly lower resting baseline mucosal TER than control subjects (*p* < 0.05; Figure 6A). We next tested the effect of increasing concentrations of lubiprostone on TER of active CD biopsies mounted in Ussing chambers. Lubiprostone induced a significant increase in TER only at the 0.1 μM concentration compared with vehicle control (DMSO only) (*p* < 0.05; Figure 6B).

### 2.6. Lubiprostone Effects on TER Are Independent of Ion Transport Stimulation

**Figure 7:**
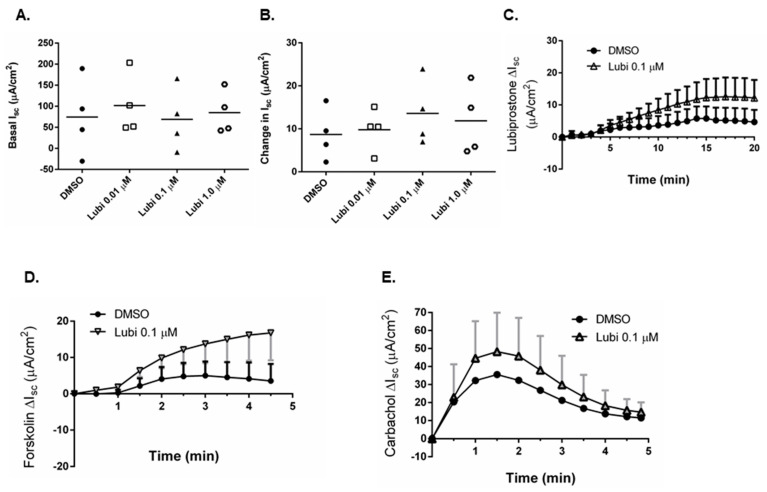
Lubiprostone improves TER independent of ion transport effects. (**A**). Baseline I_sc_ levels, expressed in μA/cm^2^, were equivalent in active CD biopsies designated for control vs. lubiprostone treatment. (**B**). Lubiprostone did not induce a significant increase in I_sc_ in active CD biopsies vs. vehicle control (DMSO). (**C**). The time course of I_sc_ responses to lubiprostone (0.1 μM) vs. vehicle in active CD biopsies revealed identical patterns of response. (**D**). Cumulative time course of the I_sc_ response to forskolin in active Crohn’s disease colonic biopsies (*n* = 4). (**E**). Cumulative time course of the I_sc_ response to carbachol in active Crohn’s disease colonic biopsies (*n* = 4).

We next investigated whether the improvement in TER induced by lubiprostone in biopsies from patients with active CD was associated with a lubiprostone-stimulated ion transport response. The baseline I_sc_ for tissues subsequently treated with either vehicle control (DMSO) or lubiprostone (0.01, 0.1, 1.0 μM) that effectively increased TER (c.f. Figure 5B) was equivalent (Figure 7A). Lubiprostone treatment of active CD biopsies mounted in Ussing chambers did not induce a significantly greater ion transport response than vehicle control, as shown by calculation of the peak ion transport response (Figure 7B) and in a representative trace (Figure 7C) of the concentration of lubiprostone (0.1 μM) that effectively increased TER (c.f. Figure 5B), despite doing so in biopsies from CD patients in remission (c.f. Figure 2B). This indicates that the ion transport machinery involved in mediating the response to lubiprostone in biopsies from CD patients in remission was desensitized or non-responsive to the same stimulus in active CD and was likely not required for the improved TER observed in Figure 5B. In addition, lubiprostone did not suppress the I_sc_ response to subsequent exposure to forskolin or carbachol (Figure 7D,E).

## 3. Discussion

Increased intestinal permeability is a well-described feature of IBD and is believed to play an essential role in the debilitating symptoms of the disease [30]. Indeed, several groups have reported that intestinal permeability is elevated in both active and quiescent disease in IBD patients [31]. Moreover, previous landmark studies have demonstrated that intestinal permeability is increased prior to development of inflammation and can also serve as a predictor of relapse in Crohn’s disease patients [32,33]. Increased intestinal permeability arising from specific modifications of epithelial tight junctions, the key structural unit regulating epithelial permeability, involves discreet and regulated changes in expression or localization of tight junction proteins such as the transmembrane protein, occludin, as described in patients with mild to moderately active IBD [34,35,36]. Our data indicate that the chloride secretory agonist, lubiprostone, may have potential value as a therapeutic agent in reducing tight junction associated permeability defects by increasing localization of the tight junction protein, occludin, in cell membranes.

While the use of an agent approved to stimulate fluid secretion, such as lubiprostone, to repair barrier function in an intestinal condition such as Crohn’s disease that is associated with diarrhea seems counterintuitive, our data indicate divergent effects of lubiprostone based on intestinal inflammation status. We observed that lubiprostone increased ion transport responsiveness (Figure 2B) and improved TER (Figure 4A) in colonic tissues from patients with CD in remission whereas in normal controls, lubiprostone increased ion transport responses but had no effect on TER. While the magnitude of the TER response is small, albeit significant, this may be due to the necessary short duration of the ex vivo experiment where tissues were exposed to lubiprostone for only 2 hrs. Moreover, in subsequent studies using biopsies from CD patients with active disease, lubiprostone also significantly improved barrier function (TER) (Figure 6B) but did not induce a significant increase in ion transport (Figure 7B,C). These experiments were restricted by extremely limited access to patients with active CD as we were only able to obtain biopsies from four patients with active CD, and in addition we were unable to perform FD4 permeability analyses. However, these data do at least provide an indication of lubiprostone efficacy in biopsies from CD patients with active disease with respect to effects on TER. Moreover, these data not only indicate that lubiprostone selectively functions as a barrier promoting agent in tissues subject to inflammation, but also indicates a functional dissociation of the ion transport vs. barrier modifying effects of lubiprostone in healthy and disease states but also between stages of disease activity. This is supported by studies in an ischemic pig model where pre-treatment of injured tissues mounted in Ussing chambers with the ClC-2 blocker, ZnCl_2_, prevented recovery of TER and occludin restoration to epithelial junctions, but only partially inhibited lubiprostone-stimulated ion transport responses [17]. This suggests that at least a portion of the ion transport response evoked by lubiprostone is not required for barrier repair and these two physiological functions are differentially activated by lubiprostone. This highly context-specific effect of lubiprostone is further emphasized by the comparatively poor responsiveness of biopsies from UC patients in remission to lubiprostone-stimulated ion transport (Figure 2B,E) coupled with a lack of effect on TER (Figure 4A). Moreover, lubiprostone improved TER but had no apparent effect on FITC-dextran permeability in biopsies from CD or UC patients although there was no significant difference in FITC-dextran permeability between biopsies from these patients and controls. While we cannot offer a definitive mechanism for the contrasting effects of lubiprostone on TER vs. FD4 permeability in CD biopsies, it is well established that these readouts of permeability are differentially regulated and involve different cohorts of tight junction, and tight junction-associated, proteins [37]. In the Caco-2 intestinal epithelial cell line, lubiprostone increased expression of the sealing claudin family member, claudin-1, and restricted IFN-g induced reductions in TER in a manner consistent with increased claudin-1 abundance [38]. Studies in a rodent water stress avoidance model show that in addition to restoring normal levels of occludin, lubiprostone can also prevent the increased protein expression of the cation transmembrane pore, claudin-2, in this stress model [39]. The same study also identified that lubiprostone could prevent claudin-2 overexpression induced by glucocorticoid treatment in intestinal epithelial cell lines and in isolated colonic crypts from healthy human subjects. This confirmed that lubiprostone inhibition of claudin-2 expression was applicable across multiple model systems. Increased claudin-2 expression is a feature of IBD and is sufficient to provoke increased paracellular leak of sodium and water. Therefore, lubiprostone inhibition of claudin-2 is consistent with an improvement in TER, and modulation of one or more claudin family members may also be a contributory factor to the observed improvement in TER in lubiprostone-treated CD biopsies in the present study. Collectively, these data also indicate that lubiprostone appears to have selective efficacy in alleviating barrier defects in CD vs. UC and also highlights an important functional difference between these disease entities. With respect to why different responses to lubiprostone were observed between CD and UC tissues, we can only speculate that either the signaling mechanisms used by lubiprostone to modify occludin are differentially affected in CD vs. UC, or there may be additional factors required to restore occludin that are more affected in UC than CD, and thus are less responsive to lubiprostone in UC.

The selectivity of the lubiprostone effect on intestinal tissue from inflamed/disease in remission vs. non-inflamed/non-disease conditions is in broad agreement with previous studies in porcine and rodent models of barrier dysfunction in experimental inflammation as well as our own previous work in control human colon comparing the effects of lubiprostone and the cGMP-activating anti-constipation drug, linaclotide [17,20,24,40,41]. Protective roles for lubiprostone have also been identified in a number of animal studies of intestinal injury caused by dextran sulfate sodium (DSS) or NSAID injury [19,29]. This was recapitulated in a prospective randomized pilot study of NSAID-induced injury, healthy volunteers were treated with diclofenac for 7 days followed by placebo or lubiprostone for 28 days [21]. Lubiprostone effectively decreased the elevated intestinal permeability (lactulose:mannitol ratio) vs. patients treated with diclofenac alone. These data further support that lubiprostone has some benefit in recovering intestinal permeability in conditions of intestinal inflammation although this particular model of diclofenac-induced intestinal permeability is likely due to epithelial injury caused by a variety of factors including prostanoid inhibition, uncoupling of oxidative phosphorylation and NSAID-induced apoptosis [18].

Our data indicate that the mechanism by which lubiprostone improves TER is associated with increased localization of occludin at epithelial junctions. This is in agreement with mechanistic studies in the aforementioned animal models investigating lubiprostone recovery of intestinal barrier function, where lubiprostone also increased membrane localization of occludin [17,20,24,40]. While we ideally would have performed corresponding Western blot analysis of lubiprostione-treated biopsies, because of the prioritization of functional electrophysiological studies we did not have biopsies available to perform further protein analysis. Moreover, we did not have IRB approval to obtain additional biopsies over and above those requested for electrophysiological and immunostaining analysis. An additional shortcoming was the lack of access to CD patients with active disease which restricted our study to four such subjects.

In summary, our data support animal model studies that the bicyclic fatty acid, lubiprostone, may selectively promote recovery of epithelial barrier properties in a disease/inflamed setting by promoting increased membrane localization of the junctional protein, occludin. The observation that lubiprostone discriminated between colonic biopsies from Crohn’s disease and ulcerative colitis patients is intriguing and further adds to the complexity of lubiprostone-induced signaling outcomes in a disease setting. Given the established heterogeneity between CD and UC with respect to location, inflammation penetrance (transmural vs. mucosal), cytokine profiles, unique vs. overlapping genetic risk variants, and most pertinently the differential responses to various treatment approaches (i.e., anti-TNF to treat CD; the pan JAK inhibitor, tofacitinib, to treat UC), it should not be surprising that lubiprostone also displayed a selective effect between IBD subtypes [42]. In animal models and human subjects, lubiprostone has been demonstrated to have additional effects on intestinal and isolated epithelial function including ion transporter trafficking, mucus release, and smooth muscle contraction [15,20,43,44]. However, our data are consistent with a beneficial role for lubiprostone in promoting the functional integrity of the intestinal mucosa, and suggest potential therapeutic efficacy of lubiprostone in improving intestinal barrier function in human Crohn’s disease.

## 4. Methods

### 4.1. Consent and Ethics Approval

Studies were performed according to the guidelines of the Declaration of Helsinki. Approval was granted by the Human Research Protections Program, University of California San Diego (IRB #071794) and written informed consent was obtained from all study subjects.

### 4.2. Patient Criteria

Control subjects were individuals referred for colonoscopy for general evaluation (anemia of unknown origin, previous diverticulitis, polyp surveillance, etc.). IBD subjects in remission had an established diagnosis of IBD for at least 3 months prior to enrollment and were referred for colonoscopy for disease assessment or surveillance. Patients were classified as being in remission based on an absence of visible inflammation by colonoscopy and with a Mayo endoscopy score of zero (UC), and a simple endoscopic score for Crohn’s Disease (SES-CD) endoscopy score of zero. Active Crohn’s disease (CD) patients had a diagnosis of CD for at least 3 months prior to the start of the study and were undergoing colonoscopy to assess disease activity. Six biopsies from macroscopically normal sigmoid colon were obtained from normal subjects undergoing routine colorectal cancer screening (*n* = 17), Crohn’s disease (CD, *n* = 16) and ulcerative colitis (UC, *n* = 15) patients in remission and CD patients with active disease (*n* = 4), by a gastroenterologist with expertise in IBD (B.J.; M.J.D.; B.S.B.) at University of California, San Diego Medical Center. Patient characteristics are included in Table 1.

### 4.3. Biopsy Collection

Colonic biopsies from sigmoid colon were obtained with large-capacity forceps (Olympus, Tokyo, Japan) and placed on gel foam inserts (Ethicon US LLC, Cincinnati, OH, USA) (mucosal side facing up) by a gastroenterologist (B.J., M.J.D, B.B.). Biopsied tissues were immediately placed into cold, pre-oxygenated Ringer’s solution (pH 7.4) with the following composition (in mM): 140 Na^+^, 5.2 K^+^, 1.2 Ca^2+^, 0.8 Mg^2+^, 119.8 Cl^−^, 25 HCO_3_^−^, 2.4 H_2_PO_4_^−^, and 10 glucose. Tissues were transported to the laboratory within 30 min of collection. A paired biopsy was placed in formalin for subsequent immunohistochemical staining

### 4.4. Immunohistochemical Staining

Immunostaining was performed on 4 mm-thick, formalin-fixed, paraffin-embedded tissue sections mounted on positively charged X-tra slides (Surgipath, Richmond, IL, USA). Paraffin sections were deparaffinized in xylene, rehydrated, and washed in H_2_O. Tissues were stained with hematoxylin and eosin to identify histological features and primarily to confirm that tissues had an intact epithelium. Images were taken with an Olympus IX71 microscope.

### 4.5. Immunofluorescence Imaging

The 4 mm-thick colonic biopsies were placed in 4% paraformaldehyde, embedded in paraffin, and sectioned (5 μM) onto positively charged X-tra slides (Surgipath, Richmond, IL, USA). Sections were de-waxed using xylene, hydrated with graded ethanol and heated for antigen retrieval (10mM sodium citrate buffer). After rehydration, an outline was drawn around the sections using a PAP pen. The sections were then treated with proteinase K (0.2 μg/mL) Tris-EDTA buffer and blocked (5% bovine serum albumin (5% BSA/PBS)).

The primary rabbit anti-occludin antibody (Invitrogen) was diluted at 1:50 in 5% BSA/PBS and pipetted onto the designated sections as a bubble held by the PAP pen outline. The slides were then incubated overnight at 4 °C in a humidifying chamber. The slides were then washed with PBS (×3), and secondary Alexa Fluor 568-conjugated goat anti-rabbit (Molecular Probes) was pipetted as a 1:100 dilution in 5% BSA/PBS onto the slides as described above in the primary step and incubated for 30 min at 37 °C to evaluate localization of occludin. The slides were washed three times with PBS. The sections were then incubated with Hoechst 33258 (Molecular Probes) in PBS (1:100) for 5 min at 37 °C and washed three times in PBS. Finally, a coverslip was mounted on the sections using ProLong Gold antifade reagent (Molecular Probes) and allowed to dry at room temperature. Imaging was performed using the Zeiss LSM 510 confocal imaging system. Occludin immunofluorescence staining of intestinal tissues was performed using ImageJ as previously [37].

### 4.6. Electrophysiological Studies of Human Colon

Mucosal biopsies were mounted on specially designed Ussing chamber inserts with a window area of 0.031 cm^2^ (Physiologic Instruments, San Diego, CA, USA). Tissues were bathed bilaterally in 5 mL oxygenated Ringer’s solution (composition as above) at 37 °C. The tissues were short-circuited by an automated multichannel voltage/current clamp (VCC MC8) and the short-circuit current (I_sc_), expressed in μA, across the tissues was monitored at intervals as an indication of net active ion transport. Tissues were allowed to equilibrate for a 30 min period, at which point baseline potential difference (PD) expressed in mV, short circuit current (I_sc_) and tissue conductance (G) were measured to confirm both tissue viability and calculate baseline mucosal transepithelial electrical resistance (TER), prior to administration of any reagents [45,46]. For longer term FITC-dextran permeability studies (120 min), tissues were maintained in open-circuit conditions apart from periodic application of current (5 mV, closed-circuit conditions) to measure tissue conductance and calculate mucosal TER.

### 4.7. FITC-Dextran Permeability Measurements

FITC-dextran 4kD (FD4) (Sigma-Aldrich, St. Louis, MO, USA; product #46944) was added to the mucosal bathing solution of colonic biopsies mounted in Ussing chambers to reach a final concentration of 2.2 mg/mL. Serosal bathing media samples were collected at 30 min intervals over a period of 120 min and an equal volume of physiological solution was added to the serosal side to negate the hydrostatic pressure imbalance. Collected samples were stored in the dark at 4 °C until fluorescence intensity (492-nm excitation and 515-nm emission wavelengths) was measured using a microplate spectrophotometer (Bio Rad, Hercules, CA, USA).

### 4.8. Pharmacologic Challenge of Human Colonic Biopsies Ex Vivo

Mucosal biopsies mounted in Ussing chambers were treated with either DMSO (0.045%) as a negative control, or increasing concentrations (0.01 µM; 0.1 µM; 1.0 µM) of lubiprostone (purchased from TLC PharmaChem, Vaughan, Ontario, Canada) on both the mucosal and serosal surfaces added to individual tissue preparations. After 120 min, tissues were treated with the cAMP-dependent agonist forskolin (20 µM; Sigma-Aldrich, St. Louis, MO, USA) applied to the mucosal and serosal side. After approximately 5 min (at the peak of the forskolin response plateau phase), tissues were treated with the Ca^2+^-dependent agonist Carbachol (300 µM; Sigma-Aldrich, St. Louis, MO, USA) to the serosal side of chamber. The concentrations of individual agonists used were based on maximally induced responses observed in intestinal tissues mounted in Ussing chambers, including ex vivo human intestine studies [47]. This approach was used to confirm both lubiprostone efficacy and tissue viability based on electrophysiological responsiveness to stimuli of electrogenic ion transport (forskolin and carbachol). Tissues that failed to respond to both forskolin and carbachol were excluded from data analysis. Data were recorded and analyzed using Labchart Pro 7 software (AD Instruments, Colorado Springs, CO, USA).

### 4.9. Data Analysis

I_sc_ responses (ΔI_sc_) to lubiprostone were calculated by subtracting the baseline I_sc_ from peak I_sc_. ΔI_sc_ responses to forskolin and carbachol were calculated in the same manner. Transepithelial electrical resistance (TER) was calculated from the conductance and I_sc_ based on Ohm’s law (R = V/I). Percent change in TER was also calculated from basal and post-treatment TER.

### 4.10. Statistics

Data are presented as the mean ± standard error of mean (SEM). Comparisons between groups were performed by using analysis of variance (ANOVA) followed by the Newman–Student–Keuls post-test or unpaired Student’s *t*-test where appropriate, using GraphPad prism software (GraphPad Software, La Jolla, CA, USA, version 5). A *p*-value of <0.05 was considered statistically significant.

## Figures and Tables

**Table 1:** Patient characteristics.

	Control	CD-Remission	UC-Remission	CD-Active
Number of patients	17	16	15	4
Sex (% female)	57	73	67	50
Age at enrollment (Years +/- SD)	61.8 ± 10.9	39.5 ± 14.3	49 ± 14.8	50 ± 14.7
**Symptoms** (**% of total population**)				
Diarrhea	7	27	13	75
Blood (rectum/stool)	7	6.7	0	25
Anemia	0	0	7	0
Abdominal pain	0	33		25
**Medical treatment** (**% of total popn.**)				
Anti-inflammatories	42.9	26.7	86.7	25
Anti-TNF	0	46.7	33	75
Steroids	0	0	6.7	25
Immunosuppressives	0	26.7	33	50

**Anti-inflammatories:** ASA, Antihistamine (Phenergan), Ibuprofen, Mesalamine (Asacol, Canasa, and Lialda), and Sulfasalazine. **Anti-TNF:** Humira and Remicade. **Steroids:** Prednisolone. **Immunosuppressives:** Azathioprine (Imuran), 6-mercaptopurine, and Tacrolimus.

## Data Availability

De-identified data are available upon request.

## Abbreviations

CD	Crohn’s Disease
CFTR	Cystic Fibrosis Transmembrane conductance Regulator
cGMP	Cyclic Guanosine Monophosphate
CIC	Chronic Idiopathic Constipation
ClC-2	Chloride Channel Type 2
DSS	Dextran Sulfate Sodium
FD4	FITC-Dextran 4kD
IBD	Inflammatory Bowel Disease
IBS-C	Constipation-Predominant Irritable Bowel Syndrome
IEC	Intestinal Epithelial Cell
I_sc_	Short-Circuit Current
TER	Transepithelial Electrical Resistance
UC	Ulcerative Colitis
